# Mitochondria fingerprint longevity in iPSCs

**DOI:** 10.18632/oncotarget.3530

**Published:** 2015-03-11

**Authors:** Andrea Masotti, Enrico Bertini, Claudia Compagnucci

**Affiliations:** Unit of Neuromuscular and Neurodegenerative Disorders, Laboratory of Molecular Medicine, Department of Neurosciences, Bambino Gesù Children's Research Hospital, IRCCS, Rome, Italy

The recent and powerful technology of the human induced pluripotent stem cells (iPSCs), (stem cells that are reprogrammed from easily obtainable adult somatic cells such as skin fibroblasts), allows for example to study the pathogenesis of still poorly known human diseases, to perform drug screenings or even to be used in translational medicine [1]. Currently many human diseases suffer from the lack of a proper animal model and cannot be studied thoroughly for the difficulty to overcome unaccesible affected tissues (i.e. neurological diseases) for further *in vitro*/*in vivo* investigations. Therefore, the iPSC system is a powerful and essential tool for those investigators engaged in understanding poorly characterized human diseases in cell models with preservation of the original genetic background. Furthermore, if properly manipulated *in vitro* the iPSCs can be differentiated into any cell type, thus allowing to analyze molecular and cellular mechanisms of cellular differentiation.

As said above, iPSCs can also be employed as a therapeutic tool (translational medicine). However, we believe that before considering the iPSC technology as an effective therapeutic option for humans, a better understanding of the patients-derived iPSCs biology is absolutely mandatory. We know that aging and cell death have been linked specifically to mitochondrial dysfunction and that cell differentiation and health requires a fine regulation of mitochondrial homeostasis. Therefore, it appears quite obvious that the ‘wellness’ of the mitochondrial environment parallels that of cells and, ultimately, individual's health. To support this hypothesis, we recall that mitophagy, the self-degradative autophagic process, eliminates harmful aged or damaged mitochondria. A growing body of evidence has indicated that removing damaged mitochondria may improve neuronal functionality and decrease myofibers degeneration in a mouse model of collagen VI muscular dystrophy [2, 3].

Indeed, an unbalanced mitochondrial recycling/elimination process through selective autophagy is considered an early event involved in the pathogenesis of other several neurological diseases such as Charcot-Marie- Tooth, Alzheimer's, Huntington's, amyotrophic lateral sclerosis, cerebral ischemic models, schizophrenia and depression. Moreover, mitochondrial function decreases with age and they both have been implicated in age-related disorders such as Parkinson's disease (PD) [4].

Therefore, to shed light on these issues, we investigated some aspects of mitochondrial aging and their effects on neuronal differentiation [5]. In particular, we compared the mitochondrial network of ‘young’ and ‘aged’ iPSCs (y- and a-iPSCs, cultured respectively one month and one year) both in self-renewal and neural differentiating conditions. a-iPSCs have a higher amount of mitochondria than y-iPSCs and when induced to differentiate, they fail to display the elongated or branched neurites otherwise observed in y-iPSCs. Mitochondria were depolarized and formed a convoluted network that occupied most of the cytoplasmic space ultimately leading cells to apoptosis. Furthermore, a-iPSCs showed an abnormal expression pattern of genes involved in mitochondrial biogenesis and functionality [5].

One significative observation outline the important relationship between mitochondrial function, aging and diseases. In fact, in the study published by Miller et al. [6], the authors managed to observe the age-related disorder of Parkinson's disease (PD) in neurons derived from iPSCs of PD patients, only after progerin over-expression by mimicking a molecular form of aging in iPSCs. Progerin expression is associated with mutations in the structural nuclear lamina protein LMNA, which causes premature aging progeroid syndromes (i.e. Hutchinson-Gilford progeria syndrome) and Progerin levels increase gradually during physiological aging. It would be interesting to check the mitochondrial network in iPSCs where progerin is over-expressed. In this regard, we can predict that mitochondrial network and function are altered, but further research is required to fully understand the causative effect of aging in this context. In conclusion, we feel that in a close future the manipulation of processes that regulate mitochondrial integrity (i.e. mitophagy and autophagy) will offer a viable and powerful therapeutic target for many neurological diseases and for all of those disorders in which the mitochondrial function is impaired and where cytoplast turnover is greatly unbalanced. Among these, the mammalian Target of Rapamycin (mTOR) pathway (known to inhibit autophagy) has been considered as a potential target for pharmacological intervention [7]. Interestingly, Rapamycin (also known as Sirolimus), which promotes autophagy, has been approved by the US Food and Drug Administration in 1999 for the treatment of cancer and its efficacy in neurological diseases would be an other example of drug repositioning. In fact, the strategy of drug repositioning consists in giving additional value to a drug by targeting disease other than those for which it was originally developed and tested (i.e. gemcitabine that was originally developed and indicated for viral infection and is currently used for cancer) and we believe that this strategy warrants further investigation.
